# Determinants and barriers of helmet use in Iranian motorcyclists: a systematic review

**DOI:** 10.5249/jivr.v9i1.890

**Published:** 2017-01

**Authors:** Homayoun Sadeghi Bazargani, Mohammad Saadati, Ramin Rezapour, Leili Abedi

**Affiliations:** ^*a*^Road Traffic Injury Research Center, Tabriz University of Medical Sciences, Tabriz, Iran.; ^*b*^Department of Health Service Management, School of Health Services Management and Medical Informatics, Tabriz University of Medical Sciences, Tabriz, Iran.

**Keywords:** Helmet, Determinants, Barriers, Facilitators

## Abstract

**Background:**

Helmet use by motorcyclists decreases the incidence and severity of an injury and its related death. Unfortunately, the helmet use rate is not in an acceptable level in Iran. This study aimed to systematically identify the determinants and barriers of helmet use among Iranian motorcyclists.

**Methods:**

A systematic search of literature was done using PubMed, Scopus, Science Direct and Web of knowledge databases for English literature and SID for Persian articles by specified keywords. Manual searching and reference of references were used to improve the articles identification. Articles published before 1995 and those which did not report the barriers and determinants of helmet use were excluded. Data were extracted using an extraction table.

**Results:**

Out of 49 retrieved articles, 13 articles were included in the study. Most of them (70%) had a cross-sectional design. Personal factors (such as older age, marital status and education) and motorcyclist's attitude and beliefs about the helmet effectiveness were reported as important determinants of helmet use. Helmet weight and its visual and audial limitation for motorcyclists were known as the main reported barriers to use a helmet.

**Conclusions:**

Interventions affecting the motorcyclists' attitude must be employed along with the legal interventions. Moreover, cost-effective engineering improvements in helmet production remain an important policy to improve the compliance of helmet use.

## Introduction

Injuries caused by traffic accidents are the major challenges within domains of public health. Actually, the case fatality rate of traffic accidents in middle-income countries compared with high-income countries was 20.1 deaths for every 100000 people while these countries had only 52% of registered vehicles.^1^ Nearly 1.24 million deaths happen every year due to traffic accidents predicted to reach up to 1.9 million cases in 2030 without performing any actions on behalf of involved organs. ^2,3^ Traffic accidents were introduced as the eighth leading cause of death in the world and were considered the first cause of death in age group of 15-29 years. ^4,5^ A study of current trends showed that road accidents will become the fifth leading cause of death in the world by 2030. ^1^ According to the World Health Organization in 2014, years of life lost (YLL) due to driving injuries between 2000 and 2012 have increased up to 14%. ^6^ In Iran, traffic injuries were considered the 3rd cause of deaths including more than 32000 deaths in 2012. Moreover, 7% of deaths of children under 5 in Iran were caused by traffic accidents in 2013.^1,4,7^ Vehicles with high vulnerability which had the highest proportion of casualties due to accidents in the world are motorcycles. Motorcyclists were the most frequent victims of accidents on urban and rural streets and put themselves and others at risk with their risky behaviors. ^8^ The probability of motor riders’ deaths during each mile (1.6 km) was 34 times more than those who use other vehicles.^9,10^ Motor riders compared to car drivers had 8 times higher risk of death, 4 times higher risk of injury, 2 times higher risk of an accident with pedestrians, and the probability of motorcycle accident is 9.3 times more than cars. ^11^ In Iran, more than 51% of transport accidents resulting in death or hospitalization happen for motor riders or passengers. ^8^ According to the World Health Organization, about 25% of victims of traffic accidents suffer from injuries related to brain trauma and the main risk factor for motorcyclists was due to not wearing helmets. Helmet use decreases the risk of death about 40 percent and severe injuries up to 70 percent in people using motorcycles. ^12,13^ Studies in Iran showed that the leading cause of death in traffic accidents was trauma to the head and neck ^14-17^ while the use of helmets in Iran is not desirable (10-43%).^18,19^

It was reported in Vietnam that only 23.1% of motorcyclists were using helmet among 94.6 percent of those carrying a helmet.^20^ Literature demonstrated that having a helmet does not necessarily lead to helmet use by motorcyclists, but people should have the intention to use helmet.^21^ In this regard, studies to identify factors affecting the use of the helmet have been carried out. Zamani et al.'s (2011) results revealed that heat inside the helmet and lack of ventilation, helmet weight, limiting the visual and audial power of drivers, disrupting the appearance of drivers, lack of maintenance place for helmet on motorcycle, and high price of standard helmet compared with non-standard helmet were the factors preventing helmet use by drivers. ^22^ Moreover, helmet heaviness, warming up inside the helmet, the length of trip, negative attitude towards the impact of helmet, existence of the mandatory law to helmet use, a person's driving experience, age of the person, type of road and type of motorcycle had been mentioned as effective factors in helmet use in other studies.^20,23-27^ Given that preventing traffic accidents and reducing related injuries were among the priorities of the World Health Organization and most countries, identifying determinants of the safety behavior of drivers especially motorcyclists in Iran could provide useful information for adopting appropriate policies. This study aimed to identify the determinants and barriers of helmet use by motorcyclists in Iran.

## Methods

**Eligibility **

Eligibility of studies to be reviewed included the articles published in peer reviewed journals, reporting determinants and barriers of helmet use, indexed in valid databases and published after 1995. Conference abstracts and articles which had reported the effects of helmet on accidents and published before 1995 were excluded.

**Identification**

Using keywords (Helmet use, predictor, determinant, affecting factor, facilitator AND Iran), literature was systematically searched in PubMed, Scopus, Science Direct and Web of knowledge databases for English literature and SID for Persian ones in 2016. Manual search of journals were done. Also, references of the selected articles were reviewed to identify more articles. 

**Selection**

Articles were first screened reviewing their titles in order to exclude non-relevant articles. Then, the abstract and full texts of the retained articles were reviewed. Endnote X5 software package was used to organize and screen articles and identify duplications. Articles quality assessment was done by two of researchers using the STROB checklist (M Saadati and L Abedi).

**Synthesis **

Extraction table was used to extract the data from the included articles. Reviewing articles and extracting information were done by two researchers (M Saadati and R Rezapour) and disagreements were solved by the third one (L Abedi). 

## Results

**Figure 1:**
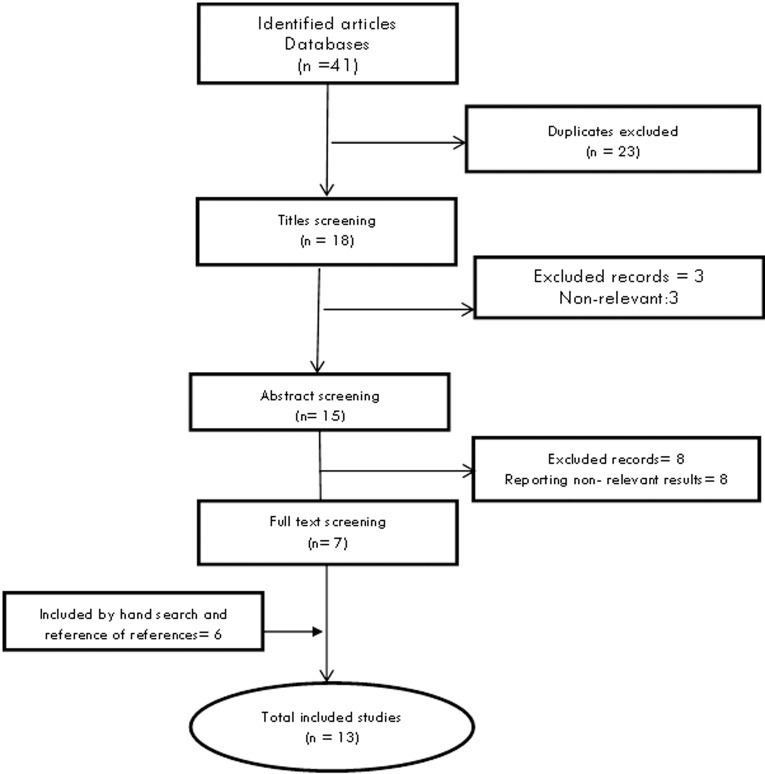
Article review and retrieval diagram

Searching articles in the databases has led to 41 articles. Moreover, 8 articles were found through hand searching. Retrieved articles were screened as reported in figure 1 , and finally 13 articles were included in the study. 

Most of the studies had a cross-sectional design (70%) and were done in Yazd, Shiraz, Kerman and Mashhad, respectively. Personal factors (such as older age, marital status and education) and motorcyclist's attitude and beliefs about the helmet effectiveness were reported as important helmet use determinants. Helmet weight and its visual and audial limitation for motorcyclists were known as the main reported barriers to use helmet. Detailed results of an individual article are presented in Table 1.

**Table 1:** Detailed information of individual included articles.

No	Authors (date)	Country/ City	Study design	Mean age	% of having license	% of wearing helmet	Barriers to wear helmet	Determinants of using helmet
1	Fereshteh Zamani et al (2011)	Tehran	mixed-method			33		• Encouragement or the positive influence of others • Being the head of the household/family. • Perceived vulnerability and severity of accident-related injury • Belief in helmet efficiency • Other perceived benefits of the helmet • Having direct and indirect experiences of traffic injury^22^
2	Mahdi Quchaniyan Haqverdi et al (2015)	Mashhad	cross sectional	31		47.6	• Feeling of heat • Decreasing vision and hearing of the rider	• Perception of social norms • Tendency to get engaged in risky traffic behaviors • Understanding the necessity and importance of using helmets • Ease of use ^27^
3	Mehri Ali et al (2011)	Iran	review	34.8		37.8		• Attitude, subjective norms, and perceived behavioral control had significant correlations with\and are significant predictors of helmet use intention.( Perceived behavioral control refers to the degree of which an individual feels that the performance of a behavior is under his or her volitional control) ^37^
4	Teamur Aghamolaei et al (2011)	Yazd	cross-sectional	26.8 (7.2)	27.6%	52%		• Perceived more behavioral control • Perceived more cues to action • More self-efficacy • Older age • Education ^38^
5	Javad Faryabi et al (2014)	Kerman	cross-sectional			21.5	• The heavy weight of helmet • Feeling of heat • Pain in the neck • Feeling of suffocation • Limitation of movement of the head and neck • Physical discomfort ^30^	
6	Seyed Taghi Heydari et al (2016)	Shiraz	cross-sectional	27	68.8%	33.1%		• Older age of rider • Married people compared with the singles • Having more driving experience • Using motorcycle for business reasons • Having license ^34^
7	Ghobad Moradi (2014)	Kurdistan	Surveillance data			5%	Low socio-economic status^39^	
8	Rezazadeh et al (2015)	Bojnourd	cross-sectional	30.06	44.6	52.8		• Married people compared with the singles • Older age of riders • Higher education • Having license • Living in city compared with village^36^
9	Ali Mehri et al (2011)	Yazd	Semi-experienced	34.7		22	• Feeling of heat • The heavyweight of helmet • Limitation in vision	• Attitude of the motor drivers • Subjective norms • Behavioral control ^37^
10	Baghianimoghadam (2010)	Yazd	cross-sectional	37.6	48.6	43.3	• Feeling of heat • The heavy weight of helmet • Limitation in vision ^19^	
11	Mokhtari et al (2015)	Kerman	cross-sectional			11.2		• Summer compared with winter • Holidays were less than other days^40^
12	Kiyarash Zinat Motlagh (2013)	Shiraz	cross-sectional	40		11.2	Uncomfortability of the helmet	• Having higher education • Using motorcycle for work ^41^
13	Abrishami et al (2014)	Mashhad	cross-sectional	30.8				• Positive attitude of helmet use • Having unsafe behaviors • Knowledge of traffic laws • More driving experience • Long time driving ^42^

## Discussion

The results demonstrated the impact of various factors including demographic ones (age, education, driving experience and marital status), attitude towards the use of helmets and some factors related to helmet (such as heaviness, feeling the heat in helmet use and helmet expensiveness) in using helmet by Iranian motorcyclists.

Proper use of the helmet reduces the severity of injury in accidents and risk of death.^9^ The results of this study indicated that the use of helmets among Iranian motorcyclists was very low (it was reported in some studies to be from 5% to 53%). The use of helmet in America was reported 60% ^28^ and in China 67.7%. ^23^ Moreover, similar statistics to Iranian studies had been reported in countries such as Vietnam and Argentina. ^20,29^ Given that more than 51 percent of traffic accidents resulted in death or hospitalization happen for motor riders or passengers in Iran,^8^ proper policies to promote a culture of using safety equipment should be taken.

Most of the helmet use barriers among Iranian motorcyclists were related to helmet problems ranging from heaviness, feeling the heat during helmet use, lack of sufficient sight of motor rider to surroundings, limiting the hearing of the rider and expensiveness of standard helmets.^19,30^ In a study by Faryabi et al. (2014), 77% of motorcyclists had raised heavy weight of helmet as the main reason for not using it followed by neck pain due to using the helmet (69.4%) and head motion restriction (59.6%), respectively, which were other important factors that have hindered the use of helmets among motor riders. ^30^ Similar results were reported in some studies in other countries such as Greece,^31^ Italy^32^ and Pakistan. ^33^ Designing a safety helmet in accordance with defined conditions of motor riders could be effective in facilitating helmet use by motorcyclists. On the other hand, studies showed that the individual factors were effective in the use of helmets. Literatures showed that, in Iran, older and married people use the helmet more than the younger and unmarried people ^34^ which was consistent with the findings of Hong et al. (2008) in Vietnam ^20^ and Kulanthayan et al. (2000) in Malaysia.^35^ Moreover, a significant relationship between higher education and helmet use was reported in other studies,^36^ which was in contradiction with the study by Heidari et al. (2015).^34^ Attitude and beliefs toward being safe and the effectiveness of helmet were issues introduced in some studies as facilitating factors for helmet use.^22,27^ Promoting knowledge and awareness of motor riders will lead to a positive attitude which will result in their positive performance. Therefore, promoting safety culture (especially helmet use) should also be taken in the priority of intervention programs in addition to the application of legal interventions.

## Conclusion

The results of this study showed that a number of different factors (individual factors, factors related to helmets and attitudes toward the use of helmets) influence the use of helmets by motor riders in Iran. In addition, individual factors such as age and marital status and other factors identified could be affected and changed by adopting right strategies. Therefore, to improve motor riders' safety culture in Iran, a comprehensive policy that covers all factors should be taken. Moreover, cheap engineering, improvements in helmets' design and production could be a good solution to improve the motorcyclists' compliance. 
